# 

**Published:** 1903-01

**Authors:** 


### PERSONALS.

Dr. Leonard Pearson, of Philadelphia, Pa., was a visitor to Boston in November in regard to some national veterinary sanitary work. Dr. Pearson was joined by Dr. James Law, of Ithaca, N. Y., and with Dr. John R. Mohler, of the Bureau of Animal Industry, Washington, D. C. An investigation proved the existence of a most serious and widespread outbreak of foot-and-mouth disease extending over four of the New England States.

Dr. William Dougherty, of Baltimore, Md., was a Philadelphia visitor in November. His advocacy of a Veterinary Assurance Society has been the means of bringing its importance very forcibly to the attention of the profession.

Dr. Leonard Pearson, State Veterinarian of Pennsylvania, addressed the Philadelphia Pathological Society in November on the subject of "Immunizing Cattle as a Measure to Protect Cattle from Tuberculosis."

Dr. Otto Noack, of Reading, Pa., was a visitor to Philadelphia, Pa., the latter part of November, calling at the JOURNAL office.

The Massachusetts Legislature for the present session has two veterinarians among its representatives. Dr. James B. Paige, of Amherst, fills one of the places for his district, and Dr. Phelps, of Methuen, who has not been in active professional work, but is a graduate of the Harvard Veterinary Department, fills a similar post.

Dr. Leonard Pearson, State Veterinarian, was a Harrisburg (Pa.) visitor early in December. The action of Governor Yates, of Illinois, in quarantining against Pennsylvania on account of foot-and-mouth disease was the source of an important meeting of the State Live-stock Sanitary Board.

Dr. William Herbert Lowe, President of the Veterinary Medical Association of New Jersey, was the guest of Dr. T. Earle Budd, of Orange, N. J., on December 22d, at the annual dinner of the New England Society in commemoration of the landing of the Pilgrim Fathers.

The many friends of Dr. George H. Berns, Brooklyn, N. Y., and those of the profession who have had the pleasure of meeting his daughter at the A. V. M. A. conventions, will be glad to learn of her assured recovery from the attack of typhoid fever that for many weeks caused her to lie at death's door.

The editor of the JOURNAL makes due acknowledgment to the "Sage of Reading" for the bountiful Christmas supply of the famous "Berks County pretzels" so generously bestowed. Not like coal "we have them to burn," and invite our friends to call around and help themselves.

Prof. A. Smith, of Toronto, Canada, in his trip abroad during the past summer met many of the profession who are active in educational and sanitary work. The schools on the other side do not seem to be enjoying as much support as those on this side of the water.

Philadelphia's milk inspectors, Drs. G. R. Hartman and F. R. Smith, continue to pursue those who expose for sale milk from unhealthy cows. In December the conviction of a dealer was had where an inflamed udder was ignored by a producer and dealer.

Dr. E. M. Ranck, of Glenolden, Pa., is a member of the Philadelphia Pathological Society. He was among those who entertained Dr. Jacobi, of New York, of the Pediatric Society of Greater New York, at their December meeting.

Dr. John J. Repp, of Ames, Iowa, is an active State association member in his State and a strong power in influencing others to be more active in association work.

Dr. Leonard Pearson, of Philadelphia, addressed the Grangers at Clearfield, Pennsylvania, early in December. Dr. Pearson has done an excellent work among those engaged in dairying and other branches of the live-stock industry in Pennsylvania.

The JOURNAL is in receipt of a beautiful calendar for the new year from Dr. H. P. Jackson, of Sewickley, Pa., sent out in connection with the livery and boarding stable maintained by our esteemed colleague.

Drs. James T. McAnulty and T. J. Kean, of Philadelphia, still continue to take an active interest in better educating the members of the horseshoers' organization in Eastern Pennsylvania.

Dr. Richard H. Powers, of Fort Walla Walla, Washington, has been transferred to Fort Riley, Kansas. Dr. Powers is a veterinarian to the artillery corps at that station.

Drs. Leonard Pearson, of Philadelphia, and E. M. Ranck, of Glenolden, Pa., were on the programme for the December meeting of the Schuylkill Valley Veterinary Medical Association.

Dr. W. L. Rhoads, of Lansdowne, Pa., takes a very active part in the Road Drivers' Association of Pennsylvania and all their plans for the establishment of "good roads."

